# Report of Alliance of International Science Organizations on Disaster Risk Reduction (ANSO-DRR) Conference 2020

**DOI:** 10.3390/ijerph17238772

**Published:** 2020-11-26

**Authors:** Emily Ying Yang Chan, Chi Shing Wong, Kevin Kei Ching Hung, Gretchen Kalonji, Peng Cui, Gordon Zhou, Rajib Shaw

**Affiliations:** 1 Collaborating Centre for Oxford University and CUHK for Disaster and Medical Humanitarian Response (CCOUC), The Chinese University of Hong Kong, Hong Kong, China; cswong@cuhk.edu.hk (C.S.W.); kevin.hung@cuhk.edu.hk (K.K.C.H.); 2 Nuffield Department of Medicine, University of Oxford, Oxford OX37BN, UK; 3 JC School of Public Health and Primary Care, The Chinese University of Hong Kong, Hong Kong, China; 4 Accident & Emergency Medicine Academic Unit, The Chinese University of Hong Kong, Hong Kong, China; 5 Institute for Disaster Management and Reconstruction, Sichuan University, Chengdu 610207, China; gretchen.kalonji@gmail.com; 6 Institute of Mountain Hazards and Environment, Chinese Academy of Sciences, Chengdu 610041, China; pengcui@imde.ac.cn (P.C.); gordon@imde.ac.cn (G.Z.); 7 Graduate School of Media and Governance, Keio University, Tokyo 252-0882, Japan; shaw@sfc.keio.ac.jp

**Keywords:** ANSO, ANSO-DRR, disaster risk reduction (DRR), Belt and Road Initiative, multidisciplinary cooperation among disaster and healthcare sciences, alliance of regional science organizations, health emergency and disaster risk management (Health-EDRM), Sendai Framework for Disaster Risk Reduction, 2030 Agenda for Sustainable Development, Paris Agreement, COVID-19

## Abstract

This article summarizes the proceedings of the four-session meeting (webinar) conducted by the Alliance of International Science Organizations on Disaster Risk Reduction (ANSO-DRR) on 18 May 2020. ANSO-DRR is an international, nonprofit and nongovernmental scientific alliance bringing together academies of science, research organizations and universities which share a strong interest in disaster risk reduction in the regions along the land-based and maritime routes of the Belt and Road Initiative. ANSO-DRR convenes an annual meeting to review its work progress and discuss its scientific programs. The first session was the opening statements and was followed by the introduction and updates on ANSO-DRR in the second session. The third session was the depiction of the big picture of ANSO, the umbrella organization of ANSO-DRR, led by the Assistant Executive Director of ANSO, while the fourth session was a presentation of perspectives on the strategic development of ANSO-DRR. One of ANSO-DRR’s key strategies is to enhance disaster mitigation and response through multidisciplinary cooperation among disaster and healthcare sciences (i.e., health emergency and disaster risk management (Health-EDRM)). It aims to enhance DRR efforts by performing as an instrument in connecting people along the Belt and Road regions, focusing on DRR resource and database development, involving higher education institutions in DRR efforts and increasing disaster resilience in built infrastructures.

## 1. Introduction

**Figure 1:**
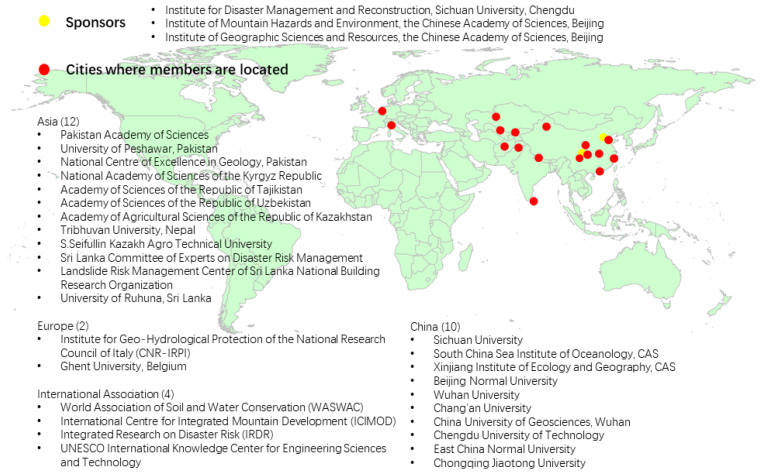
Sponsors and members of the Alliance of International Science Organizations on Disaster Risk Reduction (ANSO-DRR).

This is the report on the proceedings of the second discussion meeting (webinar) conducted by the Alliance of International Science Organizations on Disaster Risk Reduction (ANSO-DRR) on 18 May 2020. ANSO-DRR was launched during the first discussion meeting in Beijing, China on 11 May 2019, with the collaborative efforts of the Institute of Mountain Hazards and Environment of the Chinese Academy of Sciences (IMHE-CAS) [1]; the Institute for Disaster Management and Reconstruction (IDMR) of Sichuan University and The Hong Kong Polytechnic University [2]; the Institute of Geographic Sciences and Natural Resources Research (IGSNRR) of the Chinese Academy of Sciences [3]; Integrated Research on Disaster Risk (IRDR) [4]; International Centre for Integrated Mountain Development (ICIMOD) [5]; and other international scientific institutions, academies and universities from countries including China, Italy, Belgium, Tajikistan, Nepal, Pakistan and Sri Lanka which share strong interests in the field of disaster risk reduction (DRR) and a strong geographic focus on the regions along China’s Belt and Road Initiative [6]. The initiative was raised by Chinese President Xi Jinping in his visit to Central Asia and Southeast Asia in September and October 2013, and it aims to help promote the economic prosperity of the Asian, European and African countries along the Belt and Road region and regional economic cooperation; strengthen exchanges and mutual learning between different civilizations; and promote world peace and development. ANSO-DRR currently has 30 members and was formally recognized as one of the thematic alliances under the umbrella of the Alliance of International Science Organizations (ANSO) [7] on 10 December 2019. ANSO is a nonprofit, nongovernmental, international scientific organization founded in 2018 by the Chinese Academy of Sciences and 36 other international science and education institutions from around the world (see Figure 1).

ANSO-DRR aims to enhance education, young scientists’ training and international communication and to provide scientific instructions on natural disaster prevention, major infrastructure construction and socioeconomic development by collaboration among global scientists and organizations. One of its key strategies is to enhance disaster mitigation and response through multidisciplinary cooperation among disaster and healthcare sciences [8,9]. ANSO-DRR is dedicated to addressing the most urgent challenges of disaster risk reduction through collaborative efforts under the framework of ANSO. It is committed to making contributions to the implementation of the Sendai Framework for Disaster Risk Reduction 2015–2030 [10] and the implementation of the 2030 Agenda for Sustainable Development [11] at the regional level and contributing to safe, green, resilient and sustainable society through integrated efforts [12].

ANSO-DRR has identified the following key areas of opportunities to work on: DRR focusing on the countries along the land-based and maritime routes of China’s Belt and Road Initiative, building effective collaboration between participating communities, focusing on disaster resilience of infrastructures and contributing to DRR innovations in higher education and entrepreneurship.

ANSO officially reviewed ANSO-DRR’s proposal on 3 December 2019, which included the working concepts of ANSO-DRR, how the new alliance could add value to ANSO, and the work plan and target achievements for the next three years. The proposal of ANSO-DRR was accepted on 11 December 2019, and Peng Cui from CAS and Gretchen Kalonji from IDMR of Sichuan University and The Hong Kong Polytechnic University were appointed as Co-Chairs for the coming three years (2020–2023). The ANSO-DRR Secretariat is hosted at the Institute of Mountain Hazards and Environment (IMHE) of the Chinese Academy of Sciences in Chengdu, China, under the leadership of Lijun Su, Vice-Director of IMHE and ANSO-DRR Secretary-General.

## 2. Materials and Methods

ANSO-DRR convenes its annual meeting to review its work progress and its scientific programs. On 18 May 2020, ANSO-DRR hosted a meeting to review the developments of the ANSO-DRR in 2019 and discuss the future development and activities of the international organization, with the participation of more than 50 experts from 15 countries. This paper summarizes the findings from the four-session meeting. The first session comprised the opening statements. The second session was the introduction and updates on ANSO-DRR. The third session provided the background of ANSO as the umbrella organization of ANSO-DRR. The fourth session reviewed various perspectives on the strategic development of ANSO-DRR. Lastly, there were the discussion session and closing remarks by ANSO-DRR Co-Chair Cui.

## 3. Results

### 3.1. Session 1: Opening Statements

ANSO-DRR Co-Chair Kalonji noted the well-balanced membership of ANSO-DRR, with members from various relevant disciplines dedicated to solving common transdisciplinary DRR challenges, members’ nationalities well-represented along the Belt and Road regions and members having strong leadership roles in their respective institutions. ANSO-DRR Secretary-General expressed his support in organizing the webinar meeting to further promote ANSO-DRR despite the COVID-19 pandemic.

### 3.2. Introduction and Updates on ANSO-DRR

The importance of disaster risk reduction, risk management and sustainable development as foregrounded in the key international agenda for the coming decades, including the 2030 Agenda for Sustainable Development, the Sendai Framework for DRR and the Paris Agreement 2015 [13,14,15], was pointed out in the beginning. While geophysical, hydrological, meteorological, climatological and maritime hazards were widely distributed along the Belt and Road regions, many of the Asian and African countries along the regions were underdeveloped and capacities are weak in DRR. These technical constraints were exacerbated by increases in the magnitude and frequency of natural disasters as a result of climate change, which had caused dire economic loss and hindered social development in these countries [16,17]. ANSO-DRR thus aims to enhance DRR efforts by serving as a platform in connecting people along the Belt and Road regions, focusing on DRR resource development, involving higher education institutions in DRR efforts and increasing disaster resilience in built infrastructures. ANSO-DRR was introduced by Gordon Zhou of IMHE as an international, nonprofit and nongovernmental scientific alliance and a collaborative platform for sustainable development and better promoting the Sendai Framework for DRR and the 2030 Agenda for Sustainable Development along the Belt and Road regions. Currently having 30 members, it was initiated by IMHE-CAS and IDMR of Sichuan University and The Hong Kong Polytechnic University, among other global institutions sharing strong interests in the field of DRR, with its geographical focus on the Belt and Road regions. ANSO-DRR focuses on organizing meetings for leaders of various national and international organizations to exchange on latest DRR theories and practices; contributing to building up open-access, disaster-related data in and across the Belt and Road countries; promoting transnational and multidisciplinary research collaboration in DRR; aligning goals with UN Science and Technology Road Map; forging industrial partnerships; promoting DRR innovations in higher education; and providing DRR advisory support to national governments and regional intergovernmental bodies. ANSO-DRR was officially approved by ANSO on 11 December 2019. 

### 3.3. Big Picture of ANSO

Depicting the big picture of ANSO as an umbrella organization of ANSO-DRR, Likun Ai, Assistant Executive Director of the ANSO Secretariat, discussed how to promote international cooperation in science and innovation for global benefits. ANSO has 53 members in more than 40 countries, with a vision to become an international science organization of significant impact in the promotion of shared development and advances of United Nations’ Sustainable Development Goals (SDGs) through the implementation of concrete programs, initiatives and actions in science, technology, innovation and capacity building (STIC). It supports the following activities: education and capacity building; international science cooperation in areas of major importance for SDGs; international cooperation for livelihood, health and well-being; assessment for support in science policies and advice; and technology transfer. ANSO’s governance is comprised of the Presidents of Chinese Academy of Science (ANSO President), Pakistan Academy of Science (ANSO Vice-President) and Russian Academy of Science (ANSO Vice-President). Since its formal establishment in November 2018, ANSO has built up its own website and newsletter (ANSO Update) and published a strategic plan. Current ANSO programs include ANSO scholarships for young talents (200 master’s students and 300 doctoral students), ANSO training program (supporting 10 training workshops outside of China annually for ANSO members), ANSO fellowships, ANSO associations (operational arms for ANSO), ANSO collaborative research, ANSO prize (for Chinese scholars having received higher degrees from overseas to work in China) and workshops/conferences. ANSO associations aim to enhance the impact and operation of ANSO in major areas, mobilize and use resources and efforts more effectively, integrate and network scientists and units of different countries and allow easy and effective collaboration. ANSO-DRR was the second association supported by ANSO, which saw great potential for ANSO-DRR, with ANSO-DRR’s abundant resources and pool of experts. The ANSO platform could be used for ANSO-DRR to collaborate with other ANSO associations. For example, ANSO crosscutting platform aimed to establish the basic sciences, green and sustainable development, health and disease, science-based strategic advice and technology transfer by utilizing ANSO research and ANSO capacity building programs. ANSO has formulated the following targets: (1) establishing joint funding mechanism among ANSO members and partners, (2) coming up with innovative ideas on transboundary cooperation to bring in more active institutions and scientists and (3) cooperating with key international organizations at actual program level.

Two common strategic approaches of ANSO and ANSO-DRR were highlighted. The first is its commitment to transdisciplinary approaches, and the second is the focus on coupling science and technology research with capacity development. With respect to the latter, the major challenge of multinational partnerships involved a large imbalance among partners in access to resources. A model in which countries could contribute according to their capacity was called for. Mechanisms for co-funding projects were proposed: half-half, in which ANSO’s support would be based on the funding capacity of the collaborator; in-kind support for capacity building (e.g., funding local costs of meetings/trainings); and co-funding of scholarships between ANSO and partner institutes.

In response to an inquiry on the mechanism for the ANSO community to get connected, ANSO Secretariat was identified as the main platform. As to the question on the links of ANSO-DRR and the International Association for Disaster Risk Reduction (IADRR) [18], the common focuses on developing young scientists and the mapping of worldwide research activities were stressed. ANSO-DRR would also focus on clearer, more concrete and specific topics that the ANSO country members could utilize findings to similar projects (e.g. the Institute of Atmospheric Sciences used a multimodel for seasonal prediction and disseminated it to ANSO members). Regarding the question of whether topics on energy, power generation, transmission and mobile communications were covered by ANSO, it was confirmed that ANSO was preparing the energy corridor, an opportunity for collaboration in these areas.

### 3.4. Perspectives on the Strategic Development of ANSO-DRR

In this session, the importance of ANSO-DRR’s strategic development, especially in the context of the COVID-19 pandemic, were raised. Multicountry and regional collaboration, as well as transdisciplinary approaches, were highlighted. The following strengths of ANSO-DRR were also underscored: high-level participation of national academies of sciences, provision of strong opportunities for transnational collaboration, involvement of the top research institutions in the respective regions and its strong geographic focus. A challenge for ANSO-DRR was to develop a strategy for collaborating with and adding value to existing networks (e.g., the Global Alliance of Disaster Research Institutes (GADRI), the InterAcademy Partnership (IAP) and ANSO itself), rather than duplicating the work of other networks. As a result, the next goals of ANSO-DRR’s work were identified: creating better linkages between DRR communities and health emergency management (i.e., health emergency and disaster risk management (Health-EDRM)) communities [19,20], focusing on involvement of youth and young professional networks, promoting greater collaboration with engineering communities and professional societies in DRR and contributing to policy development. For ANSO-DRR to achieve these goals, a powerful knowledge platform for sharing information was called for. The need for ANSO-DRR to develop a DRR strategy in the context of COVID-19 was also emphasized. Moreover, data collection on existing mechanisms and changes in each member’s agenda in relation to COVID-19 was identified as an essential step in order to move forward.

## 4. Discussion

Following the presentation sessions, a discussion among participants was conducted on the following critical questions and issues:

- How can ANSO-DRR be promoted under the impact of COVID-19?
- Knowledge platform construction.
- Network for young scientists.
- Enlarging ANSO-DRR membership.
- Interactions between DRR community and Health-EDRM community.
- Relationship between ANSO-DRR and other relevant multinational alliances and networks.
- Expressions of interest from ANSO-DRR members in contributing to various topics.

ANSO-DRR should focus on practical solution generation to countries and communities by looking into planning tools, analytical framework and early warning mechanisms ANSO-DRR could offer to its partners. Technical scoping from ANSO-DRR partners on what their needs were should be sought.

Regarding ANSO-DRR’s platform for communication and information sharing, the online platform should host information on ongoing research activities, educational programs and scholarship and funding opportunities in the various regions and for partners in various domains. ANSO-DRR Secretariat would be responsible for this key knowledge management platform, which would be hosted at IMHE. In response to a query on open data sharing policy within the alliance, a policy for co-publication with multinational researchers would be formulated.

New ANSO-DRR initiative and collaboration should have protocols and proper documentation to facilitate communication, knowledge accumulation, and the construction of an ANSO-DRR data repository. New technology (like social media) should be used to collect public opinion on DRR.

It was suggested that ANSO-DRR should have a task team focusing on emergency response as a priority. For example, ANSO-DRR activities should be articulated to the context of COVID-19, and members were called to share information on what was currently being undertaken to address the challenges in the context of the current COVID-19 situation. There was also a recommendation for a mechanism focusing on a single priority issue each time and pooling all resources to resolve it.

The importance of ANSO-DRR having its own critical niche areas and a strategic approach to enhance partnership between academia and industries was also highlighted. For instance, youth partnership should be emphasized.

With regard to enlarging ANSO-DRR membership, the group agreed that they would warmly welcome participation by additional institutions, network and alliances worldwide that share a common interest in DRR in Belt and Road regions.

Since the first Beijing discussion meeting in May 2019, ANSO-DRR had conducted the 2019 Summer School of Silk Road Disaster Risk Reduction, participated at the Coordinators’ Meeting on Belt and Road Seismic Reduction, contributed to the 2nd International Workshop for Youth and Young Professionals in Disaster Risk Reduction Research at IDMR and kick-started the construction of the ANSO-DRR website. The planned activities of ANSO-DRR for the years 2020 to 2022 is summarized in Table 1.

## 5. Conclusions

ANSO-DRR Co-Chair Cui concluded the meeting by reiterating the compilations, reporting, mountain hazards monitoring and early warning platforms that ANSO-DRR aims to deliver in the coming three years. These would be of great use to local scientists and other DRR practitioners within the Belt and Road regions. ANSO-DRR was envisioned to become a multidisciplinary international think tank that serves as a multilateral cooperation mechanism for DRR. ANSO-DRR will have the next annual meeting in a few months’ time to review its work progress and discuss its scientific programs.

The 12 members of the ANSO-DRR Steering Committee of diverse disciplinary and geographical distribution were also endorsed in the meeting (See Appendix A). The committee is responsible for guiding ANSO-DRR in the coming years, including reviewing the scientific problems that the ANSO-DRR will work together to solve; promoting the organization to the international community; and soliciting new memberships, partnerships and financial support. The steering committee will convene twice a year to discuss key issues.

## Figures and Tables

**Table 1:** Planned work of ANSO-DRR for 2020–2022.

Year	Planned Work
2020	Publication of the Atlas of Natural Hazard Risk for the Belt and Road Publication of the Glance at the Silk Road Disaster Risk
2021	Report on the risk assessment of natural hazards along the China Pakistan Economic Corridor Mountain hazards monitoring and early warning platform Mountain hazards chain process numerical simulation and prediction platform
2022	ANSO-DRR establishment of an efficient collaboration mechanism for DRR

## Abbreviations

ANSO	Alliance of International Science Organizations
ANSO-DRR	Alliance of International Science Organizations on Disaster Risk Reduction
APSTAAG	Asia Pacific Science Technology Academic Advisory Group
CAS	Chinese Academy of Sciences
CCOUC	Collaborating Centre for Oxford University and CUHK for Disaster and Medical Humanitarian Response
DRR	Disaster Risk Reduction
ICIMOD	International Centre for Integrated Mountain Development
IDMR	Institute for Disaster Management and Reconstruction
IGSNRR	Institute of Geographic Sciences and Natural Resources Research
IMHE-CAS	Institute of Mountain Hazards and Environment of the Chinese Academy of Sciences
IRDR	Integrated Research on Disaster Risk
IRPI	Research Institute for Geo-Hydrological Protection
SDGs	Sustainable Development Goals
STIC	Science, Technology, Innovation and Capacity
UNDRR	United Nations Office for Disaster Risk Reduction
WHO H-EDRM Research Network	World Health Organization Thematic Platform for Health Emergency & Disaster Risk Management Research Network

## Appendix A

### List of the First ANSO-DRR Steering Committee Members

- Peng Cui: Academician of the CAS; Vice-President of the Chinese Society of Water and Soil Conservation.
- Gretchen Kalonji: Dean of IDMR, Sichuan University and The Hong Kong Polytechnic University.
- Qunli Han: Executive Director of IRDR.
- Ghulam Rasul: Regional Program Manager of ICIMOD.
- Alessandro Pasuto: Research Director of Italian National Research Council—Research Institute for Geo-Hydrological Protection (IRPI).
- Rajib Shaw: Executive Director of Asia Pacific Science Technology Academic Advisory Group (APSTAAG) of United Nations Office for Disaster Risk Reduction (UNDRR) and Professor of Keio University, Japan.
- Emily Ying Yang Chan: Director of the Collaborating Centre for Oxford University and CUHK for Disaster and Medical Humanitarian Response (CCOUC); Co-Chair of the World Health Organization Thematic Platform for Health Emergency & Disaster Risk Management Research Network (WHO H-EDRM Research Network); Assistant Dean and Professor of the Faculty of Medicine of The Chinese University of Hong Kong.
- Maurice Yap: Professor and former Dean, School of Health and Allied Social Sciences, The Hong Kong Polytechnic University.
- Muhammad Asif Khan: Professor of HEC Distinguished National Fellow & Member of Executive Council—Pakistan Academy of Sciences.
- Peijun Shi: President of Qinghai Normal University; Former Executive Vice-President of Beijing Normal University.
- Abdusattor S. Saidov: Vice-President of the Academy of Sciences of Republic of Tajikistan.
- Dwikorita Karnawati: Director General of the Department of Meteorology, Climatology and Geosciences, Indonesia; Former Rector of the Gadjah Mada University.
